# How prehospital emergency personnel manage ethical challenges: the importance of confidence, trust, and safety

**DOI:** 10.1186/s12910-024-01061-9

**Published:** 2024-05-18

**Authors:** Henriette Bruun, Louise Milling, Daniel Wittrock, Søren Mikkelsen, Lotte Huniche

**Affiliations:** 1 The Prehospital Research Unit, Region of Southern Denmark, Odense University Hospital, Odense, Denmark; 2 Department of Quality and Education, Ambulance Syd, the Region of Southern Denmark, Odense, Denmark; 3 The Mobile Emergency Care Unit, Department of Anaesthesiology and Intensive Care Medicine, Odense University Hospital, Odense, Denmark; 4 Department of Psychology, Faculty of Health Sciences, University of Southern Denmark, Odense, Denmark; 5 Department of Regional Health Research, Faculty of Health Science, Forensic Mental Health Research Unit Middelfart (RFM), University of Southern Denmark & Psychiatric dept, Middelfart, Denmark

**Keywords:** Ethics, Challenges, Management, Trust, Emergency, Prehospital, Qualitative research

## Abstract

**Background:**

Ethical challenges constitute an inseparable part of daily decision-making processes in all areas of healthcare. Ethical challenges are associated with moral distress that can lead to burnout. Clinical ethics support has proven useful to address and manage such challenges. This paper explores how prehospital emergency personnel manage ethical challenges. The study is part of a larger action research project to develop and test an approach to clinical ethics support that is sensitive to the context of emergency medicine.

**Methods:**

We explored ethical challenges and management strategies in three focus groups, with 15 participants in total, each attended by emergency medical technicians, paramedics, and prehospital anaesthesiologists. Focus groups were audio-recorded and transcribed verbatim. The approach to data analysis was systematic text condensation approach.

**Results:**

We stratified the management of ethical challenges into actions before, during, and after incidents. Before incidents, participants stressed the importance of mutual understandings, shared worldviews, and a supportive approach to managing emotions. During an incident, the participants employed moral perception, moral judgments, and moral actions. After an incident, the participants described sharing ethical challenges only to a limited extent as sharing was emotionally challenging, and not actively supported by workplace culture, or organisational procedures. The participants primarily managed ethical challenges informally, often using humour to cope.

**Conclusion:**

Our analysis supports and clarifies that confidence, trust, and safety in relation to colleagues, management, and the wider organisation are essential for prehospital emergency personnel to share ethical challenges and preventing moral distress turning into burnout.

## Introduction

Patients affected by acute and severe illness are dependent on emergency services manned with physically and mentally well-functioning personnel. Thus far, there has been focus on the fact that critical incidents, defined as illness or injury that threatens the patient´s survival [1], can have short-term as well as long-term negative effects on the mental health and emotional well-being of the prehospital emergency personnel [2]. In addition, several studies with focus on ethical challenges in emergency medicine [3–12] have recently elucidated the complex and challenging clinical context in which the work of prehospital emergency personnel takes place. In this context, moral distress has received increased attention during the past decades. In 1984 Jameton introduced the concept of moral distress in nursing as resulting from situations “when one knows the right thing to do, but institutional constraints make it nearly impossible to pursue the right course of action” [13]. Later studies including other health care professionals [14] have contributed to a broader understanding of moral distress. Morley et al. [15], in their narrative synthesis of the literature, defined moral distress as the presence of a causal relationship between the experience of a moral event and the experience of psychological distress. Psychological distress is described as the presence of psychological and/or physical reactions. These may include feelings like anxiety, frustration, guilt, anger, sadness, psychological exhaustion, helplessness, and depression. Physical symptoms may include insomnia, nausea, migraines, abdominal pain, tearfulness, and physical exhaustion. As in other areas of healthcare moral distress has been described in emergency medicine [16]. Moral distress is recognised as a serious problem among nurses, physicians, and other healthcare professionals as it negatively affects them as individuals, as well as affecting their relations with patients, relatives, colleagues, and external collaborators. Moral distress is associated with burnout and the inclination to leave employment in healthcare [15, 17].

To meet these challenges some health care institutions provide clinical ethics support services to assist healthcare professionals in managing ethical challenges and reduce the risk of burnout from moral distress. Although research into clinical ethics support is relatively new, literature reviews evaluating specific ethics support services like clinical ethics committees [18], moral case deliberation [19, 20] and clinical ethics consultation [21] have been published. To our knowledge, little is known of clinical ethics support in prehospital emergency medicine.

### The purpose of the research project

This study is part of a larger action research project carried out in collaboration with prehospital emergency personnel in the region of Southern Denmark [3]. The overall purpose is to develop and test an approach to clinical ethics support that considers the context of emergency medicine and the local settings. This purpose is supported by a grounding in practice philosophy and empirical ethics [22, 23]. In this paper, we report on how prehospital emergency personnel manage day to day ethical challenges. In a previous paper [3], we reported on how ethical challenges were experienced by prehospital emergency personnel.

Method.

### The Danish emergency services

The prehospital system in Denmark is three-tiered. The basic resource is an ambulance manned by two emergency medical technicians (EMT) or paramedics (PM) [3, 24]. Following a caller’s contact with the emergency dispatch centre, the healthcare dispatcher, commonly a nurse, a PM, or an EMT, manages the call and dispatches one or more of the following units: an ambulance, an ambulance and a rapid response paramedic unit, an ambulance and an anaesthesiologist-manned mobile emergency care unit (MECU), or an anaesthesiogist-manned helicopter service. MECUs are dispatched in approximately one case in four alongside an ambulance using a rendezvous model [11]. The dispatchers’ choice of ground-based or helicopter-based supplementary unit depends on the geographical location of the incident or patient and is primarily based on the estimated response time of the unit in question. In the region of Southern Denmark ground-based rapid response units carry out the vast majority of missions requiring a supplemental unit.

### Research design

Action research pertaining to interventions in health care constitutes the overall methodological approach of this research project [25, 26]. Action research is practice-based, sensitive to organisational context, and offers a relevant research strategy when aiming to identify, develop, and test context-sensitive forms of clinical ethics support in prehospital emergency medicine. By actively involving prehospital emergency personnel throughout the process, it is likely that barriers to the development of clinical ethics support can be identified and addressed. The research project [3] is divided into three parts:

1. Identification of experiences with and management of ethical challenges in emergency medicine.
2. Collaborative development of an approach to clinical ethics support in emergency medicine.
3. Testing of the approach.

The authors of this paper formed the core research group. For the investigation of ethical challenges, and the development and testing of various forms of clinical ethics support the researchers were affiliated with the regional prehospital research group, and collaborated with anaesthesiologists, EMTs and PMs from the emergency medical system of the Region of Southern Denmark. Further, ad hoc collaboration was established with a prehospital communications officer and prehospital educators in discussions of research activities, data analysis, communication, and the planning and testing of a context-sensitive approach to clinical ethics support [3].

### Data collection

The results reported in this paper are based on three semi-structured focus groups conducted with EMTs, PMs, and anaesthesiologists [3]. Focus groups were chosen to elicit narrative data based on social interaction amongst participants. The aim was to gain insight into a variety of ethically challenging situations along with clinical reasoning and social negotiations around the best course of action for the patient [27, 28]. During focus groups, participants compared and commented on statements prepared to facilitate discussion. The focus groups generated insights into experiences, management strategies, reflections, and contrasting views among colleagues on the topic of ethical challenges [3]. The participants were recruited through an internal information network. In this local setting, most ambulance crew teamed up with the same partner during most shifts. The majority of the anaesthesiologists had been manned the local MECUs since the inception in 2006 and on average their prehospital workload amounted to two to five monthly 24-hour shifts. In total, 15 EMTs, PMs, and MECU physicians participated in the three focus groups [3].

Table 1 [3] shows the distribution of professional background and gender of the participants in the focus groups. On average the nine EMTs and PMs had been employed in a prehospital services for 15.3 years, ranging from 6 to 28 years. The six MECU physicians had been employed in a prehospital service for an average of 13.9 years, ranging from 4 to 30 years.

**Table 1:** Focus group participants

	Prehospital emergency personnel	
	MECU physicians	EMT and PM
Focus group 1	2 males, O female	2. males, O female
Focus group 2	1 male,l female	4 males, O female
Focus group 3	1 male, 1 female	2 males, 1 female
Total	4 males, 2 females	8 males, 1 female

Each focus group lasted two hours and were conducted in a meeting room at the prehospital unit [3]. Participants and facilitators were all facing each other. The focus groups took place during the late hours of the afternoon and sandwiches and soft drinks were provided. Authors HB and LH facilitated all three focus groups, while author LM participated as an observer and assisted by collecting written consent forms and managing audio recordings. As part of the focus group process one facilitator took notes on a flip chart in plain view for all to comment on. Authors SM and DW did not participate in the focus groups in any capacity due to their respective positions as lead consultant of the MECU and head of the department of quality and education. Three overall questions guided the focus groups [3]:

1. When and what kind of ethical challenges do you experience in your work?
2. How do you manage these ethical challenges?
3. In what ways does your workplace provide support for managing ethical challenges?

Audio files were transcribed verbatim. During transcription and further processing of data, any names of persons and places mentioned during the focus groups were anonymised. Interview transcripts and photographs of the flip charts were stored on a secure server.

### Data analyses

Data was transferred to and systematised in NVivo (QSR International, Burlington, Massachusetts, USA). The analytic procedure was guided by systematic text condensation [29]. As data proved rich in descriptions of both experience with and management of ethical challenges, the research team decided to focus on each separately. First, the analysis emphasised the experience of ethical challenges in day to day work [3]. Second, the analysis was focussed on the management of ethical challenges and how personnel interact with colleagues and external collaborators in the context of the emergency services.

The process of analysis was iterative involving meaning condensation of each transcript followed by moving back and forth between the condensed meaning units and theoretical concepts. To analyse how prehospital emergency personnel managed ethical challenges during incidents, we drew on the concept of moral conduct coined by Vetlesens and Nortvedt [30, 31]. In particular, we drew on the idea of a three-part sequence of *moral conduct* initiated by *moral perception*, followed by *moral judgment*, leading to *moral action*. According to Vetlesens and Nortvedt [30, 31], moral perception is a precondition for moral judgement and rests on the individual being open to the world, and receptive to the events that take place in it. Moral judgement encompasses the interpretation, understanding, and balancing of the welfare, interests, and rights of the parties concerned. The ability to perceive what is morally significant and to feel affected by a situation that affects the well-being of others is an expression of the individual’s capacity for empathy. Empathy is thus an important aspect of moral conduct.

## Results

Inspired by Avraham et al. [1] the analysis of prehospital emergency personnel´s management of ethical challenges are divided into activities before, during and after specific incidents. Interview extracts are presented avoiding redundant utterances.

1. Before incidents.

### Mutual understandings and shared worldviews

The interpersonal environment of the local ambulance station is important to how prehospital emergency personnel engage in specific incidents and how they manage ethical challenges. The participants in the focus groups discussed the development of “mutual understandings” and “shared worldviews” as a basis for working together in ethically challenging situations. Several participants described how they distanced themselves from prehospital emergency personnel at other stations for example if these seemed more concerned with “getting home to bed” than doing what was best for the patient. Another example would be if colleagues spoke or acted disrespectful when dealing with patients.

### Managing emotions

The way emotions are viewed and handled is important not just to the personal wellbeing, but also in relation to how difficult situations may be handled. Prehospital emergency personnel engage in emotionally demanding jobs and face human suffering, tragedy, and death daily. Having to pay attention, make decisions, and act in ethically challenging situations adds emotional strain. Several participants in the focus groups described how their attitudes towards the mutual expectations of “the right way” to deal with their emotions at work had changed over time.

In the old days, [ ] we were told if you can’t handle the sight of someone smashed in a traffic accident, then you shouldn’t be here [ ]. It’s not like that today. We can say I actually cannot handle this. I think that was terrible, I would really just like some help to get past it. (Text example 1)

EMTs and PMs pointed to more training and the employment of more female personnel as reasons for these changes. Several participants explained how they routinely talked with colleagues about emotional reactions to incidents nowadays. They also conveyed generally caring about the well-being of each other and being involved in each other’s private lives more so now than they used to. These changes in attitudes towards emotions at work were described as related to changes in how the job was now perceived and recognised as emotionally demanding.

My father often said, he could not understand how I could remain in this job, because as he said, you are so soft. [ ] I thought a lot about that, [ ] my answer has become that I can remain in this job because and not in spite of me being as soft as I am. So, in essence,, [ ] if I need to cry over something, well then I just do it. (Text example 2)

Some pointed out that this changed perception and recognition of their job as emotionally demanding would sometimes be at odds with the public expectation of swift and unaffected action in any situation, as well as with the public perception of them as heroes setting aside their safety to help patients. From the perspective of EMTs, PMs, and MECU physicians, heroes are unsafe and foolhardy. Conversely, they view themselves as well-considered professionals who pay attention to guidelines and struggle with what to do when the guidelines are contrary to what they consider to be in the best interest of the patient.

2. During incidents.

### Moral perception

The focus groups offer many examples of the moral perceptions involved in end-of-life incidents. In the example below, a PM conveys his awareness of signs that indicate the patient’s state of health relative to the consequences of further professional action.

Now, we are not the ones to make the final decision [concerning resuscitation], but it may take ten minutes for the MECU to arrive, and until then you try to get as good a picture of this patient [ ] as possible. It may be the nursing home resident with all kinds of ailments, who is still warm [without obvious signs of death], and where you [according to legislations] should actually start [resuscitation]. It may well be that you quickly manage to call [the MECU to have the physician order you to refrain from resuscitation], (Text example 3)

Another PM explained trying to gather as much information about the patient as possible to aid decision-making further down the line, including decisions that would prove ethically challenging. Several participants described how they would try to get an understanding of the patient, for example by looking for clues around the house, e.g. a pre-packed box of palliative medication. Discovering and developing an understanding of the patient is part of what it means to perceive morally relevant aspects of a situation.

### Moral judgment

The focus group participants reported bringing various resources into play when they reflect on what would be the best possible action in specific situations. The physicians in particular referred to *bioethical concepts* such as paternalism and autonomy, e.g. when considering whether an individual patient was competent to make their own decisions. One physician said he acted largely in accordance with ontological ethics based on the idea of human interdependence [32–34], while other colleagues reported being guided by utilitarian principles.

Several participants referred to non-specific *gut feelings.* Such sensory-emotional evaluations receive little attention and are not recognised as valuable resources for moral judgement the way cognitive-linguistic evaluations are. In one focus group, a PM explained how he relied on his gut feeling to guide his classification of a patient as either competent or incompetent, and from that decided on relevant actions in the specific situation.

It is very much up to one’s individual gut feeling. And I mean, it’s just nice to have the instructions and the legislation too, and I also think it’s nice to have the MECU to back you up. I may have a gut feeling but then you can always just call the MECU and be either confirmed or be told, no, we will just transport him [to hospital]. (Text example 4)

A physician drew attention to his *personal moral judgement* of particular patients such as rapists or violent offenders and reflected on his difficulties in empathising with all patients equally.

One is not that empathetic. That is, it’s not necessarily substandard, but it may well be that [the patient] doesn’t get the whole package of empathy. Sometimes you can be challenged by someone who is so unsympathetic or outright repulsive that you have a difficult time delivering what you really ought to. (Text example 5)

Further, participants discussed how their *moral judgment changed* through their work with different patients over time.

Who really decides whether a life is worth living. I have personally had my boundaries moved a lot after I started working at the centre for chronic respiratory insufficiency. We treat people who may be paralysed from head to toe but still insists that life is good and worth living. (Text example 6)

### Moral action

The focus groups convey that EMTs, PMs, and physicians have different tasks and roles, and therefore different room for manoeuvring when faced with ethical challenges.

### Moral action – EMTs and PMs

EMTs and PMs commonly work independently at the scene and engage in moral perception, judgement, and action on many occasions. Amongst the EMTs and PMs of the focus groups, it is not uncommon to request support from a physician. Sometimes the physicians are consulted because EMTs or PMs “need their backs covered”, sometimes they look for “an assessment of available options”, or “help to settle a disagreement”. In the following example, a PM contacted a physician because he wanted to test his gut feeling.

My partner [in the ambulance] and I are faced with some decisions to make… We do not always agree on the decisions and sometimes we agree to disagree and need the extra support or the extra point of view [ ]. We need to call and discuss a patient [with the MECU]. (Text example 7)

Sometimes EMTs and PMs expect physicians to contribute to their moral actions in decisive ways. As they are subject to the physicians’ decisions, it is important for them to have confidence in the physicians´ professional competences, whether they agree with them or not. Asked how he feels if a physician interrupts his treatment of a patient with cardiac arrest and orders it stopped, an EMT replied:

It depends on the situation, but [ ] in the cases I’ve been involved in, it has really helped a lot. Especially when we’re dealing with old people with dementia and [ ] cancer [ ] fortunately sometimes “an adult”, a physician, arrives, who can say stop, stop, stop [the futile treatment]. That, to me, is terrific. (Text example 8)

However, sometimes EMTs and PMs feel overruled. Particularly if the physicians do not make their reasons explicit or do not take the considerations of EMTs or PMs into account before taking action.

EMT 7: It’s obvious, if you’ve made a decision and you’re going one way, and then the MECU arrives, for example, and turn your decision around. To some extent, it is OK, but it can make you feel a little bit inadequate, feel that I didn’t do it well enough.

MECU 6: Yes, the issue of over-ruling [is not nice].

EMT 7: Yes, and that’s exactly why it’s important to be open and say, well, based on this and that, we’ll make this decision, it is about including each other’s opinion (Text example 9).

One EMT explained how various strategies were employed to influence the physicians to take the kinds of actions that a paramedic would prefer. Moreover, both EMTs, PMs and physicians mentioned how collaboration depended upon good working relations. Therefore, working with familiar staff is important. Several EMTs and PMs mentioned that if disagreements arise, they would try to point it out and initiate a conversation about it. Asked how they do this, they replied:

EMT1: In plain Danish.

MECU2: Hands off! [the patient]

EMT1: Is it worth it? [to treat this patient]

MECU2: Are you sure that’s a good idea?

EMT1: Yes, don’t you t’ink it’s a good idea?

MECU1: No, I’m discarding the adrenaline from the syringe (Text example 10).

Sometimes the EMTs and PMs do not find it appropriate to question or attempt to influence a physician’s decision in front of a patient or their relative as this can potentially undermine the patient’s feeling of being safe and well taken care of. On occasions, EMTs or PMs disagree with a physician to an extent where they see no other option than to withdraw from the situation altogether. They do so by turning to legitimate practical tasks unrelated to the treatment of the patient, e.g. “turning the ambulance around in the driveway”.

### Moral action – medical physicians

Although knowing the thoughts and considerations of EMTs and PMs are generally not the physicians’ top priority, some are conscious of actively involving EMTs and PMs in the decision-making process.

If you must terminate cardiopulmonary resuscitation and make the call that this is futile treatment, I usually look around at the team and then ask, do we agree that this is futile? I know that the final decision rests on my shoulders, so to speak, but still, there may be someone in the team who either finds it very surprising that we should stop or have some information, which might change my position [towards continued treatment]. (Text example 11)

In addition, physicians are aware that their decisions and actions, including their personal conduct, has an impact on how an incident is managed overall. If a physician is considered acting in an unprofessional, unreasonable, or ethically questionable manner, it could affect the entire team and their actions. During the focus groups physicians commonly staged their professional competencies as the starting point for assessing the optimal action in a specific situation.

Professionalism is hugely important because it provides a firm standpoint. It should not be necessary to talk about whether what we are doing out there is good enough because we are acting optimally according to the present conditions (Text example 12).

Several physicians stressed that it was important to them to be busy with treatment-related tasks such as intubation or peripheral intravenous cannulation at the scene and to manage the technical aspects well. However, as the following text example illustrates, some physicians recognised that their “obsession” with technical solutions did not always bring about the best possible result.

I was summoned to an old woman who had a foreign body in her throat [ ]. We carry with us something called a Magill forceps, which we can use to pry out foreign bodies from the throat and then we save lives. The relatives were standing outside crying, and I thought, wow, that patient is someone’s lovely grandmother. We must do everything we can to help her. I removed the foreign body that had caused the cardiac arrest. It was relatively simple. I then went outside the room and told the relatives that now we had saved the life of the patient. Then they cried even louder. It turned out to be the nursing staff who were crying with relief that this tormented person had now finally found peace. And then you think, oops, that it would have been nice if you had known that in advance, and maybe you had “hurried slowly”, right? (Text example 13)

Moreover, physicians are aware of and pay attention to the legal implications of specific clinical assessments.

After all, it is a medical assessment whether you think the person is competent or not competent, and if you have decided that the patient is competent, then it is straightforward in terms of legislation, you cannot grab him and abduct him into an ambulance. And then you might have some thoughts that it would be best if he went to the hospital and stuff like that, but you just can’t do that. (Text example 14)

In addition to having the patient’s best interest at heart, physicians’ actions are shaped by legal requirements. Physicians are quite aware that what is legally warranted may not always be what is best for the patient, and the risk of a complaint plays a role in their decision-making.

If you’re a [prehospital] physician and [ ] you admit everyone, your ass is never on the line. There will never be a complaint. No relatives complain that their relative has been admitted to hospital. Isn’t that just fantastic! But they don’t understand what it means to be hospitalised when you are 90 years old, confused, and has dementia [ ] I sometimes have a bad taste in my mouth about some of the things I do. [ ] Not because I’m lazy, but it just… maybe I’m a bit lazy, I don’t want any complaints, I am not interested in sticking my neck out like that (Text example 15).

3. After incidents.

### Ethical challenges are difficult to share

The focus group participants recognise that they encounter a wide array of ethical challenges on the job [3]. They pointed out that the medical implications and practicalities of an incident is often discussed, whereas ethical implications for several reasons are not.

First, ethical challenges are *emotionally taxing*. As described above, moral perception, judgement, and action engage health professionals not just reflectively but also emotionally. Participants explained that ethically challenging situations can trigger thoughts and emotions for a long time after the incident. Some conveyed that *doubt and uncertainty* can linger about whether they have done the right thing in a given situation. Others described a sense of *frustration* in situations where they cannot do what they consider right. Several participants pointed out that ethical challenges can evoke feelings of *incompetence and guilt*. To share and discuss ethical challenges can be emotionally as well as morally unsettling. Furthermore, in the context of modern-day healthcare, some participants expressed *fear of repercussions* should they be found “guilty” of acting in ways considered unethical. Some participants conveyed a general awareness of the possibility of *having errors exposed publicly* in the press or on social media. For one participant this awareness has turned into a *fear that his “whole life could disintegrate”* if he does not act appropriately in an ethically challenging situation. He referred to a specific situation where a colleague risked overlooking a broken leg of a woman covered head to toe in religious clothing out of respect for her integrity. It would amount to medical misconduct not to find and treat the leg.

Let’s take the example that “X” brought up about some burka-clad woman sitting in a car involved in an accident. There are rules and regulations that we can’t talk our way around. If I don’t discover that the patient has a broken leg, then I can lose my livelihood, I can lose my reputation, and my well-being… my whole life can be dissolved. (Text example 16)

This description points to how the fear of mismanaging an ethically challenging situation is linked to the fear of not just compromising your personal and professional integrity, but of being publicly exposed, and risking your job. Such fears and worries are not conducive to sharing thoughts or emotions related to ethical challenges in the workplace.

Second, participants reported examples of *organisational barriers*. One such is the experience of EMTs and PMs of *being corrected* by someone in charge if they “bend the rules slightly” in the best interest of the patient and to be able to live with their own actions.

EMT1: In the past we could, well, say there’s rigor, that’s fine, we’ll stop right here.

MECU: Well, the legislation has been the same all these years. We just had a practice of bending the rules a little so that we could live with it. However, every time someone [up higher] discovered it, it was like [audible smack of hands] only doctors are allowed to terminate treatment [when there are no obvious signs of death], but you just think the patient is dead, by all means [terminate treatment]. Nevertheless, it’s got something to do with people’s sense of security. (Text example 17)

Another barrier is the lack of a forum with *“*organisational impunity*”* for EMTs and PMs to discuss difficult ethical challenges. All participants recognised that the use of debriefing after critical incidents has become more common in recent times and offers an occasional chance to touch on emotional reactions to ethically challenging situations. However, this is coincidental rather than intended. Likewise, turning to psychological treatment after traumatic events or to address mental health issues has become more widespread and acceptable, but is still the exception rather than the rule.

But I’ve been there, where I received some psychological treatment and stuff like that. It is fantastic. And it probably also means that you can manage a little longer, if you can look a little inwards and say, stop, I guess I need a little help here. (Text example 18)

Rather than sharing, a widespread way of processing ethically and hence emotionally, challenging incidents is the use of dark humour amongst colleagues, “when no one else is around”.

We really have a sick sense of humor, and I was glad we were absolved from our sins by a crisis psychologist, who once said, that he recommended dehumanisation sometimes. That the use of grotesque words to talk about grotesque experiences is actually okay. And not a sign that we are emotionally detached as long as we are able to act professionally and with empathy. (Text example 19)

A few participants shared ethical challenges with a partner, close relative, or friend, in particular if these were also healthcare professionals. Several commented that they would never burden their private relations with the horrors of work.

Now that my partner is not a healthcare professional, I cannot come and share these considerations [with her]. She would think I was part of a crazy world where people die and there are bombs, explosions everywhere, and things like that. At times when I’ve had a girlfriend or a partner who was a nurse, I could also share considerations [at home] within the family. (Text example 20)

One participant conveyed that he finds relief in a religious belief when faced with the ethical challenges of working with life and death on a daily basis.

If we can help the patient and the patient survives, then his number wasn’t up. Should the patient die even though we did everything we could, well then it was his turn, his number had been drawn and that’s how it is. (Text example 21)

### Professional confidence, interpersonal trust, and organisational safety

The focus group participants gave examples of how they share and discuss ethical challenges with colleagues. They pointed out that sharing is important in maintaining long-term mental health.

But I think those of us who make it, we are good at talking to colleagues at work too. It is not always conscious; it’s just a need we have to talk. (Text example 22)

Several focus group members said that ethically sensitive conversations mostly take place in *informal settings*, such as in the ambulance or MECU on route back to the station after an assignment. It was important to the participants that they had *confidence* in their conversational partner. Confidence was inspired by medical skills and experience. Newly educated or newly recruited colleagues were not the first choice for sharing thoughts or emotions around ethically challenging incidents. In addition, it was of utmost importance that participants felt they could *trust* the person to respond with care and integrity. This was raised in the context of physicians discussing ethical challenges with EMTs or PMs.

Yes, but the better you know your paramedic at the MECU [ ] the better the communication. You dare to stand up for yourself, but you also dare to articulate what the problems are, dare to articulate that you may need some help. You should not underestimate the possibilities that working 24-hour shifts with the same man gives you. In other words, if you open up yourself, you get something back. (Text example 23)

Equally, confidence and trust were important for EMTs and PMs, who discussed ethical challenges with physicians.

Then you also dare to ask afterwards, that was really weird. What was this about? In other words, we use our doctors a lot [ ]. You [the physicians] are asked about both large and small matters subsequently, right, [ ] and it is usually to learn something for the next time you find yourself in a similar situation. (Text example 24)

Additionally, physicians pointed out that they have the opportunity to discuss ethical challenges at mandatory monthly group meetings for the MECU physicians.

So we talk about it at the monthly meeting for MECU physicians. There we have a forum [ ] and opportunity to report cases where, I for example, had been clumsy, or what have you, or: What would you have done in a similar case? (Text example 25)

This forum was portrayed as providing confidentiality and a *safe space* for discussing actions that could classify as mistakes.

Yes, yes, exactly, but then opting out of treatment is discussed and made explicit, it becomes like, legitimate. Sometimes we even have it in writing that this patient should not receive standard treatment because the person in question has abused the system any number of times. (Text example 26)

## Discussion

### Professional confidence, interpersonal trust, and organizational safety

Faced with ethical challenges, the moral conduct of prehospital emergency personnel requires moral perception and moral judgment to arrive at moral action. Further, the management of ethical challenges hinges on taking the requirements and demands of the prehospital emergency services and, when applicable, the external collaborators into account. When ethical challenges arise, they are often accompanied by emotions such as doubt, insecurity, inadequacy, and guilt. One participant expressed fear of losing his livelihood, reputation, and well-being in certain circumstances (text example 16). Some participants talked of individual management strategies, such as turning to religious or spiritual thought (text example 21) or sharing with a partner, close relative, or friend (text example 20). The use of dark humour is an integral part of how colleagues generally interact when outsiders are not present (text example 19). When prehospital emergency personnel involve colleagues in a dialogue on ethical challenges, confidence in their professional experience and competence is fundamental, just as interpersonal trust is a prerequisite for sharing. Professional confidence and interpersonal trust is built up over time and through regular collaboration (text examples 23 and 24).

The literature on inter-collegial trust in healthcare is limited. One example is Calnan and Rowe who, in their book “Trust Matters in Healthcare” [35], write about trust among clinicians. The authors find that medical competence is central when clinicians build trust in each other. However, being technically skilled is not sufficient. Interpersonal values like confidentiality, honesty, reliability, and good manners are equally important. In addition, the clinician must act in the patient’s best interest. Earlier trust among clinicians was achieved through hierarchical systems, but today, so the authors claim, trust is built and maintained over time. The assessment of confidentiality, honesty, reliability, and medical competence is an ongoing process. Further, the authors describe how clinicians can lose confidence in a colleague if their medical competencies are brought into serious question. Minor flaws are accepted. More importantly, confidence can be jeopardised if someone fails to show respect for a colleague. The authors find that a low level of trust causes a lack of confidence and increased criticism that in turn perpetuates the lack of trust within a team. A high level of trust leads to openness, better communication, and effective working relationships.

In our study, the prehospital emergency personnel predominantly point to informal forums as the context were ethical challenges are discussed. They report that they deliberate on ethically challenging situations in the ambulance or MECU on route back to the unit´s base. There are several reasons why prehospital emergency personnel do not discuss ethical challenges more broadly in the organisation. EMTs and PMs do not appreciate a sense of being publicly corrected (text example 17) and they do not have a forum with organisational impunity. Medical physicians are invited to present and deliberate on incidents that turned out in unwanted or unintended ways at monthly group meetings (text example 25). Although a younger physician expressed hesitation, as he did not feel confident enough to present his ethical challenges, several other physicians voiced their appreciation of this organisational opportunity to share and found it useful (text example 26).

Our study indicates that prehospital emergency personnel need to feel confident in and to trust their colleagues and managers if they are to share the ethical challenges they experience at work. French et al. [8] describe that emergency medical service professionals prefer to discuss ethical conflicts with peers, friends, family, and union delegates rather than following the formal organisational procedures (turning to a superior). This is because people who are not representatives of the organisation are viewed as more empathetic and can be consulted without fear of the legal repercussions that could follow from a formal process. Thus, a lack of trust is the primary reason for them not to engage with organisational processes or supporting committees. Calnan and Rowe [36] provide a general description of what characterises trust relations between clinicians and managers. In contrast to trust between clinicians, which is largely based on medical competence, trust in managers is driven by honesty and accessibility, but also to the extent to which they act in the interests of the clinical practice. Clinicians lose confidence in their managers if they appear to prioritise meeting government targets over clinical needs. Clinicians distrust managers “if their involvement in running service was seen as interference with clinical decision-making and indicated a lack of respect for clinicians’ professional judgement and autonomy. “Distrust was created particularly when clinicians felt managers were interfering to save money but this results in poorer patient care” [36]p137. Trust, on the other hand, is reflected in clinicians’ desire to share confidential ethical challenges related to patient care with their managers.

### Professional background, moral judgement, and possibilities of action

In addition to legal requirements, medical guidelines, and demands from external collaborators, the professional background of prehospital emergency personnel influence moral perception, judgement, and action in specific incidents. Although the purpose of our study was not to investigate differences between physicians, EMTs, and PMs we found that physicians pay attention to what they can and must do medically and technically (text example 12, 13), to avoid complaints and court cases more so than EMTs and PMs. Further, physicians include theoretical perspectives to a larger extent when they reason about their actions.

Several studies indicate that there are differences in the ethical reasoning of different healthcare professionals. A survey based on 2129 respondents conducted by Telleus et al. [37] showed that caregivers like nurses more often assess ethical challenges in a relational position while physicians more often take a deontological position. Concurrently, Telleus et al. [37] discuss that although some empirical studies have been conducted on ethical decision-making processes among healthcare professionals most studies are theory driven. One exception is a study by Agledahl et al. [38] based on participant observation among 15 physicians from different medical specialities. The authors describe how physicians handle ethical challenges in clinical settings. Across medical specialities, physicians approach ethical challenges in a relatively uniform way. They break down the patient’s history, amplify the patient’s complaints, and categorise them according to medical symptoms. Focus is directed at the patient’s functional level and existential aspects remain unexplored.

Another exception is an interview study conducted by Hurst et al. [39] investigating physicians’ handling of ethical challenges. The authors interviewed internal medicine physicians, oncologists, and physicians in intensive care units about the ethical challenges they had experienced and how they acted. The authors found that when physicians are confronted with an ethical challenge, they seek assistance and try to avoid a conflict, protect their own integrity, conscience and reputation, and protect the group of people involved in the decision and their integrity.

In our study, we found that prehospital emergency personnel base moral judgment on the perception of morally relevant information and clues in the environment. EMTs and PMs described relying on their gut feeling (what we have termed sensory-emotional evaluation) and on physicians’ assessment of the situation. Further, physicians pointed to clinical guidelines, legislation, and bioethical concepts. To our knowledge, there is no literature analysing the process of moral judgment among EMTs and PMs. Goethals et al. [40] have described moral reasoning and behaviour among nurses in a literature review. Nurses’ ethical reasoning is a complex process based on moral theories, ethical principles, and situational aspects embedded in the specific context of the nurse-patient relationship. Ethical reasoning emerges from the patient’s need for care and is influenced by the nurse’s relationship with the patient’s relatives and the team in the clinical context. The authors conclude that the ethical behaviour of nurses is closely tied to relational and contextual aspects of care.

In our study, EMTs and PMs are legally obliged to answer to physicians’ decisions. It can prove difficult for EMTs and PMs to act against their own moral judgement when they do not agree with physicians. Some try to indirectly influence the physicians towards the desired sequence of events or comment directly on physicians’ choices (text example 10). Sometimes EMTs and PMs refrain from getting involved if overt disagreement is deemed irreconcilable with good patient care. For some EMTs and PMs, at times, the only solution can, be to leave the scene in order not to take on responsibility for actions they disagree with and to demonstrate their disapproval. Physicians, on the other hand, are aware that they are accountable to medical guidelines and legislation, as well as being responsible for the team collaboration to achieve the best possible outcome for the patient.

Our and other studies describe how the considerations of healthcare professionals are influenced by their working conditions and medical disciplines (somatic [41, 42], psychiatry [43, 44], primary health care [45, 46]), as well as their position and responsibility (nurses [14], physicians [47, 48]). However, our study clarifies an interdisciplinary perspective in moral conduct that stresses the asymmetrical power relationship between physicians, EMTs and PMs. Consequently, EMTs and PMs discreetly try to influence physicians’ decisions. If that is not possible, they may withdraw from direct engagement in patient care. EMTs and PMs convey that this is motivated by their commitment to protect patients and relatives from experiencing disagreement or conflict between the prehospital emergency personnel in a distressing situation. Further, they do not want to jeopardize their own professional reputation or risk hampering future interdisciplinary collaboration. Overall, the EMTs and PMs weigh their professional integrity against protecting patients and securing future working relations with physicians. Articulating ethical challenges that bring the asymmetric power relations into question may have major personal and professional consequences for the individual EMT or PM. Therefore, these cases are delicate, and those who articulate them are vulnerable. Paradoxically, asymmetric power relations is one key barrier to clinical ethics support [49], while at the same time offering a framework for structured dialogue on the consequences of power relations amongst prehospital emergency personnel [19].

### Personal capacity for empathy as essential for moral conduct

According to Vetlesen and Nordvedt [30], moral conduct is a result of the emotional-cognitive process of moral perception and judgement resulting in action. Fundamental to any moral conduct is the ability to be receptive to the moral significance of a specific situation, and how the situation affects the well-being of the people involved. “Emotions are active and indeed indispensable in disclosing to us that others’ weal and woe is somehow at stake in a given situation” [31]. The individual’s emotional life resonates with his or her ability to empathise with others. The ability to empathise is developed exactly through growing up with others. Moral conduct in emergency services, as elsewhere, is based on the perception of what is morally significant, which is ultimately a result of the entire personal biography, including experiences at work. The personally developed capacity for empathy is a foundation for doing the job and is challenged, developed, or hampered on the job. The growing acceptance of psychological treatment (text example 18) may come with the risk that challenges associated with moral behaviour are privatised and individualised. The relevant and natural psychological reactions prehospital emergency personnel experience because ethical challenges may even be seen as pathological. Even if individual psychological treatment can provide an opportunity to reflect on work-related ethical challenges, it does not support organisationally grounded and collective ways of developing strategies for managing ethical challenges. This contrasts with generalising and normalising the emotional ability that forms the basis of moral perception, which is fundamental for prehospital emergency personnel to make decisions based on a moral assessment of a specific situation. Privatisation hinders an important dialogue about the psychological protection and well-being of personnel who are expected - and consider it an important part of the job - to empathise with their patients.

### Establishing clinical ethics support

Moral case deliberation [50]is one of several different ways of organising clinical ethics support. In a literature review, Haan et al. [19] describe the impact of moral case deliberation in healthcare settings. The authors identify four thematic clusters: (a) changes that are brought about on a personal and inter-professional level, concerning the healthcare professional’s feelings of relief, relatedness and confidence; understanding of the perspectives of colleagues, one’s own perspective and the moral issue at stake; and awareness of the moral dimension of one’s work and of the importance of reflection; (b) changes that are brought about in caring for patients and families; and (c) changes that are brought about on an organizational level. Moreover, the authors identify a cluster of themes concerning (d) facilitators and barriers in the preparation and context of MCD, i.e., a safe and open atmosphere created by a facilitator, a concrete case, commitment of participants, a focus on the moral dimension, and a supportive organisation.

Traditionally, professionals embedded in a medical culture are reluctant to share ethical challenges with outsiders, as they can be seen as being disloyal to colleagues [51]. The inclination to share ethical challenges differs between various healthcare professionals. While nurses find it very important to share their ethical dilemmas and decisions with other nurses and to receive support [40], physicians are more reluctant to do so [49]. Yet our analysis supports other studies [14] showing that moral conduct – involving moral perception, judgement, and action – is rarely understood or acted on by health professionals as a simple matter of right or wrong. By contrast, ethical challenges are commonly staged as complex situations involving decisions and actions dependent on legal, medical, and organisational demands [3, 8]. For these reasons, individual coping strategies are not enough to address moral distress. The development and testing of context sensitive forms of clinical ethics support in the prehospital emergency services of the region of Southern Denmark will be discussed elsewhere.

### Strength and limitations

The results of the study are based on data generated using mixed focus groups with the participation of both MECU physicians, EMTs and PMs. Focus groups may offer a safe place for participants to reflect on topics that are difficult to talk about, e.g. because of stigmatization or taboo. By including MECU physicians, EMTs and PMs we have ensured sufficient dynamics in the focus group for different experiences, perspectives and coping strategies to be articulated. However, the presence of MECU physicians in the focus group could have had the consequence that not all EMTs and PMs experienced the focus group as a safe place, which is why they may have withheld important and relevant perspectives. Nevertheless, a rich and diverse data material was collected and it became clear that there were different areas of responsibility and opportunities for moral action depending on whether it was MECU physicians or EMTs and PMs who experienced ethical challenges.

A limitation concerning external validation to other healthcare branches is that generally, in healthcare services, female employees are overrepresented. In Denmark, however, in the prehospital emergency system, the personnel traditionally consists mostly of male employees. Although this trend is slowly changing, the gender distribution in the focus groups reflects the gender distribution at present.

Moreover, the participants were recruited through information about the purpose of the study. This may have resulted in an overrepresentation of prehospital emergency personal who were particularly interested in ethical challenges. By not necessarily forming a representative sample of prehospital personnel, this study cannot quantify the overall incidence of ethical challenges among prehospital emergency personnel.

Despite the limitations described above, our comprehensive descriptions of the context of the research project may enable readers from other parts of the healthcare system to assess differences and apply our findings with relation to their clinical practice. We thus have sought to enhance the transferability of the study´s results through a reader-based analytical validity.

## Conclusion

Ethical challenges were accompanied by emotions such as doubt, insecurity, inadequacy, and guilt. The prehospital emergency personnel employed informal management strategies, such as talk on route back to the station or during breaks, sharing with a partner, close relative, or friend, but mainly if they were health professionals, or in many cases, the use of dark humour. Our analysis supports and clarifies that confidence, trust, and safety in relation to both colleagues and management are essential for prehospital emergency personnel to share ethical challenges. Thus, preventing moral distress turning into burnout or the inclination to leave employment in healthcare is closely associated with skills and experience of colleagues, the extent of interpersonal trust, and with organisational support and safety.

## Data Availability

Individuals may be identified from audio files. In adherence with the regulations of the Danish Data Protection Agency they are thus not available for public distribution. The pseudonomised transcriptions of the audio files (in Danish) are available from the corresponding author on reasonable request.

## Abbreviations

EMT: Emergency medical technician
MECU: Mobile emergency care unit
PM: Paramedic

## References

[1] Avraham N, Goldblatt H, Yafe E (2014). Paramedics’ experiences and coping strategies when encountering critical incidents. Qual Health Res.

[2] Oliveira AC et al. Working in prehospital emergency contexts: stress, coping and support from the perspective of ambulance personnel. Int J Workplace Health Manage, 2019.

[3] Bruun H (2022). Author correction: ethical challenges experienced by prehospital emergency personnel: a practice-based model of analysis. BMC Med Ethics.

[4] Milling L (2021). Documentation of ethically relevant information in out-of-hospital resuscitation is rare: a Danish nationwide observational study of 16,495 out-of-hospital cardiac arrests. BMC Med Ethics.

[5] Milling L, Lassen AT, Mikkelsen S (2021). Transparency in out-of-hospital cardiac arrest resuscitation: decision-making when patients are in the grey area between treatment and futility. Eur J Emerg Med.

[6] Brochner AC et al. Does the morning morality effect apply to Prehospital anaesthesiologists? An investigation into diurnal changes in ethical Behaviour. Healthc (Basel), 2020. 8(2). 10.3390/healthcare8020101 PMC7349197 32316371

[7] Bijani M (2021). Major challenges and barriers in clinical decision-making as perceived by emergency medical services personnel: a qualitative content analysis. BMC Emerg Med.

[8] French E, Casali GL. Ethics in emergency medical services–who cares? An exploratory analysis from Australia. EJBO Electron J Bus Ethics Organ Stud, 2008. 13.

[9] Torabi M (2020). Barriers to ethical decision-making for pre-hospital care professionals. Nurs Ethics.

[10] Torabi M (2019). Ethical decision-making based on field assessment: the experiences of prehospital personnel. Nurs Ethics.

[11] Mikkelsen S (2017). Characteristics and prognoses of patients treated by an anaesthesiologist-manned prehospital emergency care unit. A retrospective cohort study. BMJ open.

[12] Sandman L, Nordmark A (2006). Ethical conflicts in prehospital emergency care. Nurs Ethics.

[13] Jameton A (2017). What Moral distress in nursing history could suggest about the future of Health Care. AMA J Ethics.

[14] Kälvemark S, et al. Living with conflicts-ethical dilemmas and moral distress in the health care system. Volume 58. Social science & medicine; 2004. pp. 1075–84. 6. 10.1016/s0277-9536(03)00279-x 14723903

[15] Morley G (2019). What is ‘moral distress’? A narrative synthesis of the literature. Nurs Ethics.

[16] Trautmann J (2015). Relationships among moral distress, level of practice independence, and intent to leave of nurse practitioners in emergency departments: results from a national survey. Adv Emerg Nurs J.

[17] Burston AS, Tuckett AG (2013). Moral distress in nursing: contributing factors, outcomes and interventions. Nurs Ethics.

[18] Crico C (2021). Evaluating the effectiveness of clinical ethics committees: a systematic review. Med Health Care Philos.

[19] Haan MM (2018). Impact of moral case deliberation in healthcare settings: a literature review. BMC Med Ethics.

[20] Kok N (2023). Effect of Structural Moral Case Deliberation on burnout symptoms, Moral Distress, and Team Climate in ICU professionals: a parallel cluster Randomized Trial. Crit Care Med.

[21] Bell JAH (2022). Clinical ethics consultations: a scoping review of reported outcomes. BMC Med Ethics.

[22] Vogel S. What is the philosophy of Praxis? Crit Theory Thought Andrew Feenberg, 2017: p. 17–45.

[23] Musschenga AW (2005). Empirical ethics, context-sensitivity, and contextualism. J Med Philos.

[24] Mikkelsen S, Lassen AT (2020). The Danish prehospital system. Eur J Emerg Med.

[25] McCormack B. *Action research for the implementation of complex interventions*, in *Complex interventions in health*. Routledge; 2015. pp. 326–37.

[26] Malterud K (1995). Action research–a strategy for evaluation of medical interventions. Fam Pract.

[27] Halkier B (2010). Focus groups as social enactments: integrating interaction and content in the analysis of focus group data. Qualitative Res.

[28] Halkier B. *Practice theoretically inspired focus groups: Socially recognizable performativity?* A New Era in Focus Group Research: Challenges, Innovation and Practice, 2017: pp. 389–410.

[29] Malterud K (2012). Systematic text condensation: a strategy for qualitative analysis. Scand J Public Health.

[30] Vetlesen A, Nortvedt P. Følelser Og moral. (sensitivity and moral). Hans Reitzels forlag; 1997.

[31] Vetlesen AJ. *The perseption of the Moral*, in *perception, empathy, and judgment: an inquiry into the preconditions of moral performance*. Penn State; 2012.

[32] Løgstrup KE. *Den etiske fordring (English translation: Løgstrup, K.E. 1997)*. Vol. 2. 1956, 1991, Denmark: Gyldendal.

[33] Løgstrup KE (1997). The ethical demand.

[34] Rabjerg B (2017). Løgstrup´s Ontological Ethics An analysis of human interdependent existence. Res Cogitans.

[35] Calnan M, Rowe R. *Trust between clinicians*, in *Trust Matters in Health Care*. 2008, McGraw-Hill Education (UK). pp. 84–119.

[36] Calnan M, Rowe R. *Trust relations between clinicians, managers and patients*, in *Trust Matters in Health Care*. 2008, McGraw-Hill Education (UK). pp. 122–147.

[37] Telléus PK, Holdgaard DM, Thørring B (2018). Physicians and caregivers do differ in ethical attitudes to daily clinical practice. Clin Ethics.

[38] Agledahl KM, Forde R, Wifstad A (2010). Clinical essentialising: a qualitative study of doctors’ medical and moral practice. Med Health Care Philos.

[39] Hurst SA, Hull SC, DuVal G, Danis M (2005). How physicians face ethical difficulties: a qualitative analysis. J Med Ethics.

[40] Goethals S, Gastmans C, de Casterle BD (2010). Nurses’ ethical reasoning and behaviour: a literature review. Int J Nurs Stud.

[41] Reiter-Theil S, Schürmann. The big five in 100 clinical Ethics Consultation cases. Bioethica Forum, 2016. 9.

[42] Forde R, Vandvik IH (2005). Clinical ethics, information, and communication: review of 31 cases from a clinical ethics committee. J Med Ethics.

[43] Molewijk B, Engerdahl IS, Pedersen R. Two years of moral case deliberation on the use of coercion in mental health care: which ethical challenges are being discussed by health care professionals? Clin Ethics, 2016. 11.

[44] Bruun H (2018). Ethical challenges assessed in the clinical ethics Committee of Psychiatry in the region of Southern Denmark in 2010–2015: a qualitative content analyses. BMC Med Ethics.

[45] Lillemoen L, Pedersen R (2013). Ethical challenges and how to develop ethics support in primary health care. Nurs Ethics.

[46] van der Dam S (2014). Ethics support in institutional elderly care: a review of the literature. J Med Ethics.

[47] Bringedal B (2018). Between professional values, social regulations and patient preferences: medical doctors’ perceptions of ethical dilemmas. J Med Ethics.

[48] Hurst SA (2007). Ethical difficulties in clinical practice: experiences of European doctors. J Med Ethics.

[49] Bruun H (2019). Implementing ethics reflection groups in hospitals: an action research study evaluating barriers and promotors. BMC Med Ethics.

[50] Molewijk B (2011). Clinical Ethics Committee Case 13: should the school doctor contact the mother of a 17-year-old girl who has expressed suicidal thoughts?. Clin Ethics.

[51] Pedersen R, Akre V, Forde R (2009). Barriers and challenges in clinical ethics consultations: the experiences of nine clinical ethics committees. Bioethics.

